# A clinico-radiographic and histomorphometric analysis of alveolar ridge preservation using calcium phosphosilicate, PRF, and collagen plug

**DOI:** 10.1186/s40902-019-0215-3

**Published:** 2019-09-02

**Authors:** Tarun Kumar AB, Chaitra N. T., Gayatri Divya PS, M. G. Triveni, Dhoom Singh Mehta

**Affiliations:** 1 Department of Periodontics, Bapuji Dental College and Hospital, Davangere, Karnataka 577004 India; 2 Happy smiles Dental Care, Vidyanagar, Davangere, Karnataka 577004 India; 3 Consultant Periodontist, Guntur, Andhra Pradesh 522006 India

**Keywords:** Alveolar ridge preservation, Atraumatic extraction, Calcium phosphosilicate, Histomorphometry, Implant

## Abstract

**Background:**

Tooth extraction commonly leads to loss of residual alveolar ridge, thus compromising the room available for the implant placement. To combat the post-extraction alveolar loss, alveolar ridge preservation is practiced, with the advent of the biomaterial available. The purpose of this study was to assess the efficiency of calcium phosphosilicate biomaterial in alveolar ridge preservation. Twenty patients indicated for extraction were selected followed by socket grafting using calcium phosphosilicate. Implant placement was done 6 months postoperatively during which a core was harvested from the preserved sockets. Clinico-radiographic measurements of hard and soft tissues were taken at baseline and 6 months post-grafting.

**Results:**

There were no significant changes in the radiographic and soft tissue parameters while significant changes in hard tissue parameters with 1.9 mm (*p* = 0.013) gain in mid-buccal aspect and 1.1 mm (*p* = 0.019) loss in horizontal bone width were observed. The histomorphometric evaluation depicted the vital bone volume of 54.5 ± 16.76%, non-mineralized tissue 43.50 ± 15.80%, and residual material 2.00 ± 3.37%.

**Conclusion:**

The implants placed in these preserved ridges presented 100% success rate with acceptable stability after a 1-year follow-up, concluding calcium phosphosilicate is a predictable biomaterial in alveolar ridge preservation.

## Background

The predictability of dental implants was increased with the revolutionary phenomenon of osseointegration. A major emphasis is now over the three-dimensionally ideal placement of the implant for the efficient function of the restoration to be placed. The most acceptable and desirable position of the implant would be in the alveolar socket itself to mimic the natural dentition [1]. Alveolar process undergoes disuse atrophy after extraction. Schropp stated that the post-extraction healing and residual ridge dimensions are likely to be dependent on the alveolar crestal bone levels at the extraction site rather than that of the adjacent tooth [2]. Hence, to prevent the loss of residual ridge, atraumatic extraction is indicated without flap reflection. Preservation of the extraction socket by socket grafting would aid in minimal post-extraction resorption for prosthetically driven implant placement into the previous extraction socket [3]. Benex extractor is one such system used for atraumatic exodontia [4].

Socket preservation is a technique which aids in reducing the post-extraction dimensional changes in alveolar bone [5]. The biomaterial or combination of materials with anticipated regeneration capacity is invited for ridge augmentation procedures. Among the plethora of biomaterials available, alloplasts, the synthetic bone substitutes are widely accepted due to less patient morbidity, no demand for a secondary surgical site, reduced graft rejection, etc. Calcium phosphosilicate is one such alloplastic material which is easy to manipulate, readily packable, and a good hemostatic properties. Thus, this biomaterial was selected for preserving the extraction sockets.

Platelet-rich fibrin (PRF) and collagen plug were used as an adjunctive for the graft adopting the principles of guided bone regeneration [6, 7]. This study aims at clinical and histological evaluation of a traumatically extracted and grafted socket with calcium phosphosilicate putty enriched with PRF and collagen plug, 6 months after grafting. Further predictability of the grafted socket was done, evaluating the stability quotients of the implants and radiographic assessment of the crestal bone levels around the implant.

## Materials and methods

**Fig. 1:**
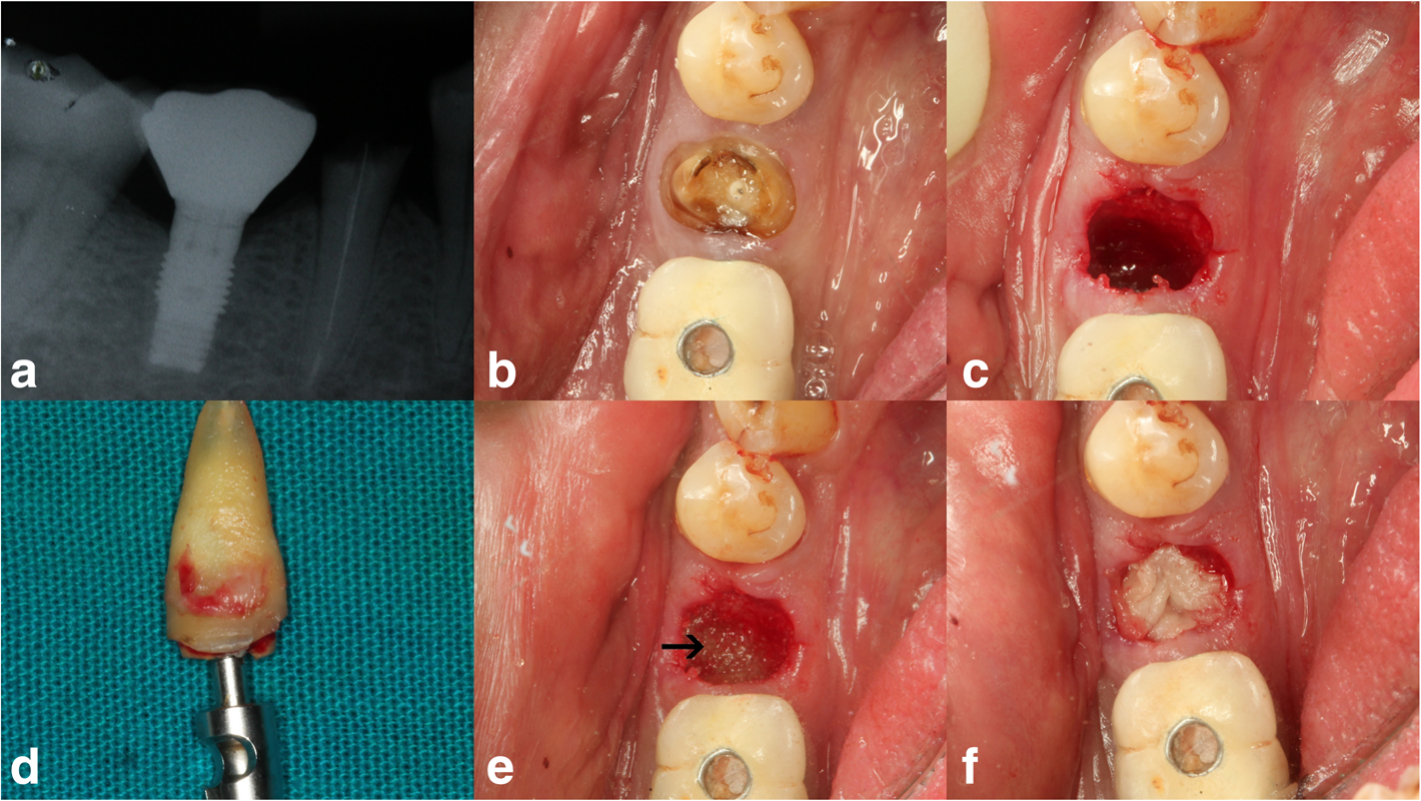
Pre-operative radiographic (**a**) and clinical representation of premolar indicated for extraction (**b**). Atraumatic extraction done (**c**) using the Benex extraction system (**d**) followed by socket grafting with calcium phosphosilicate biomaterial (**e** pointed using arrow) and PRF placement (**f**)

This longitudinal prospective interventional clinical study was conducted on 20 (8 male and 12 female) healthy subjects within the age range of 18–50 years, with hopeless teeth indicated for extraction (Fig. 1a, b).

Inclusion criteria for selection in the present study considered the following conditions which were indicated for extraction like

- Root fractures
- Endodontic failures
- Caries
- Internal root resorption
- External root resorption
- Tooth with open apex
- Over-retained deciduous tooth

**Fig. 2:**
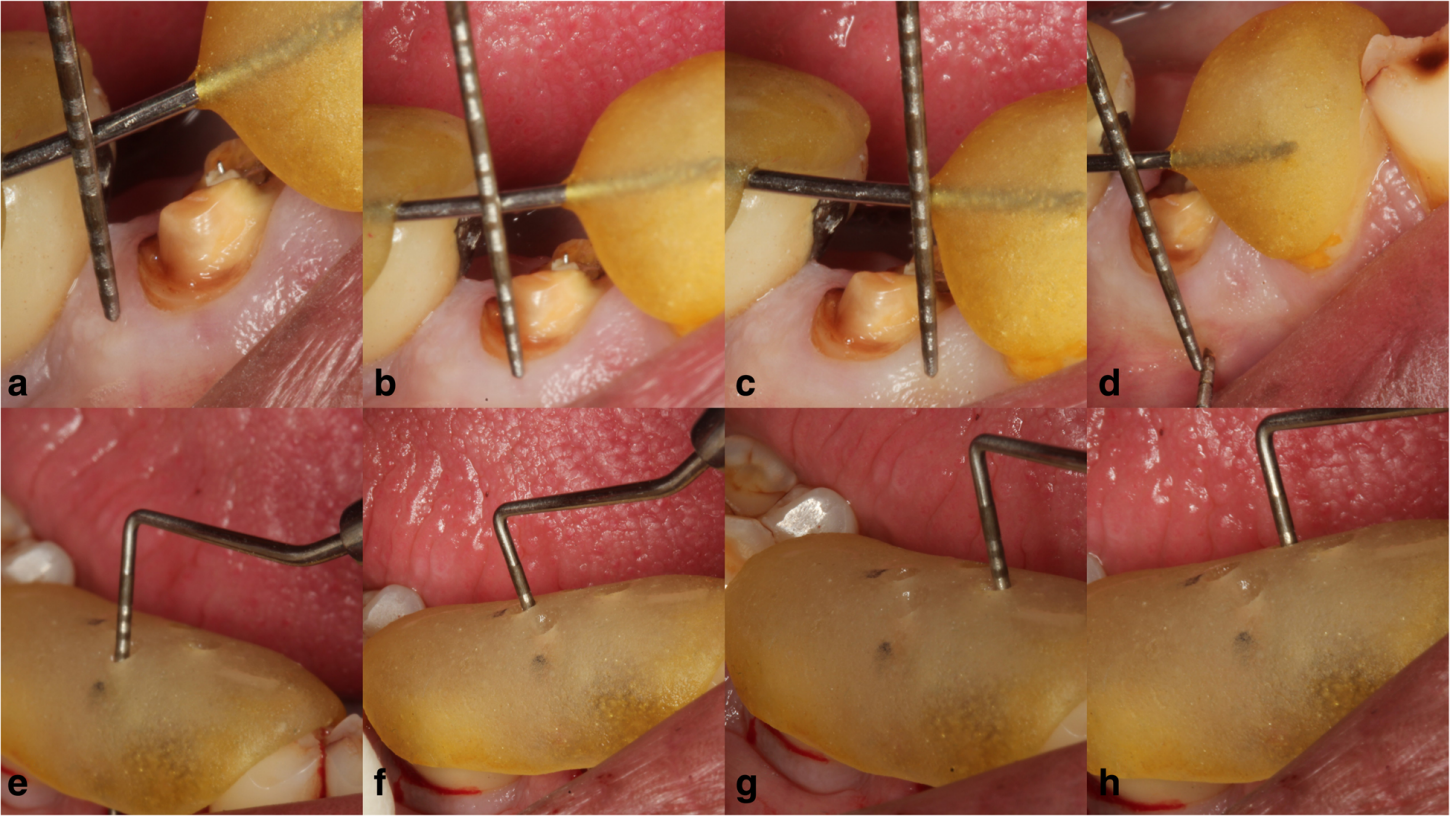
Measurement of keratinized gingival height distal (**a**), mid-buccal (**b**), mesial (**c**), and width of keratinized gingiva (**d**) using stent. A different stent with access holes fabricated was used for marginal bone measurement in mid-buccal (**e**), mid-distal (**f**), mid-mesial (**g**), and mid-lingual (**h**) locations following the extraction

The study protocol was commenced after obtaining approval from the institutional review board according to the principles of the World Medical Health Association Declaration of Helsinki 2000 for medical research in human subjects. A detailed medical and dental history with required laboratory blood investigations was done. It was made sure that all the patients included in the study belonged to ASA class I physical status. Orthopantamographs (OPG) and intraoral periapical radiographs of the area of interest were taken. Diagnostic casts were made, and customized acrylic stents were fabricated for standardized measurement of soft tissue parameters and marginal bone height levels at the baseline and postoperatively. Clinical parameters like the following were measured at the baseline pre-operatively and 6 months postoperative:

- Gingival index (Loe and Sillness) [8],
- Plaque index (Sillness and Loe) [9],
- Marginal bone levels at four sites (mid-buccal, mid-palatal, mid-mesial, and mid-distal) using the stent [9], (Fig. 2e–h)
- The bucco-palatal/bucco-lingual width of the socket [10],
- Keratinized mucosa index (Cox and Zarb) [11],
- Papillary height measurement using stent [12]. (Fig. 2a–d)

Oral prophylaxis was done 4 weeks before the extraction, and instructions were given on oral hygiene maintenance and its significance on the treatment prognosis. Patients were given one capsule of Augmentin 625 mg 12 h before and the same dose was given along with Ketorolac 10 mg 1 h before the procedure. One tablet of Alprazolam one night before was considered for apprehensive patients.

### Surgical procedure

On the day of extraction, firstly, PRF was prepared using Choukron’s protocol. Two percent Xylocaine HCl with adrenaline 1:80,000, crevicular, or sulcular incisions were placed around the indicated tooth with No: 15C Bard-Parker blade. Periotome followed by the Benex system was used for extraction (Fig. 1c, d). The extraction socket was thoroughly irrigated and curetted, followed by an exploration of the extraction socket using UNC-15 probe for fenestration or dehiscence on the buccal plate, if any. Socket grafting was then done using calcium phosphosilicate (Novabone® putty) graft, which was directly delivered to the extraction socket with the cartridge (Fig. 1e). The graft material was packed in 3–4 bulk amounts until the extraction sockets were completely filled to the height of the alveolar crest followed by PRF placement over the graft (Fig. 1f). Then, a resorbable collagen plug (ACE Surgical Supply Co., Inc.) was placed over the PRF, and mattress sutures (5–0 polyamide, Trulon, Sutures India, India) were given which were removed after 1 week. The following post-surgical instructions and medication were given:

- Cap. Augmentin 625 mg was given twice and Tab. Ketorol thrice daily for 5 days.
- Rinse mouth gently using an oral rinse (Chlorhexidine gluconate) twice daily for 15 days.
- Patients were prohibited from chewing or putting any kind of load in the surgical area.
- The patient was asked to apply an ice pack over the operated area intermittently for the first 12 h after the surgery.
- In case prolonged bleeding persisted, patients were asked to report to the hospital.

There was no evidence of any complication during the course of study. The clinical parameters, recorded at the baseline, were re-recorded for the full mouth as well as for the selected sites, at 6 months after extraction using the same stent to standardize the measurements and minimize the error. Only 16 patients (6 males, 10 females) returned for the follow-up examinations at 6 months. The plaque index and gingival index were taken for the full mouth, while keratinized mucosa index, soft tissue height, and marginal bone levels were recorded at the study site at baseline and 6-month intervals.

A bone core was harvested from the center of the socket using a trephine bur during implant placement. Further evaluation of the grafted socket was done by studying the implant stability using the Osstell ISQ system and marginal bone around the implant. Stability was measured at the implant placement and 4 months later, during loading [13]. The alveolar crestal bone levels of the implants were measured during the loading and 1-year post-loading using intraoral periapical radiographs. All the radiographs were taken by a single operator using the long-cone technique to minimize the inter-operator variability in the radiographic measurements.

### Outcome measures

The primary outcome measures of this interventional prospective clinical study are to study the:

- Soft tissue profile in grafted sites baseline and 6 months after socket grafting
- Marginal crestal bone changes in grafted sites clinically 6 months post-grafting
- Patterns of bone regeneration in the grafted sites histologically after 6 months of socket grafting

#### Histomorphometric analysis

Harvested cores were dehydrated with a graded series of alcohols for 9 days. Following dehydration, the specimens were infiltrated with a light-curing embedding resin (Technovit 7200 VLC, Heraeus Kulzer, Wehrheim, Germany) and were then ground into thin longitudinal sections in apicocoronal direction. The ground sections were stained with Stevenel Blue and Von Gieson fuchsin stain and observed under polarized light microscope at low (× 10), medium (× 20), high (× 40), and very high magnifications (× 100).

The secondary outcome measures are:

- Stability of implants placed in the grafted socket using resonance frequency analysis during the placement of the implant and 4 months postoperatively
- Changes in the crestal bone height during the implant placement, loading (4 months postoperatively), and 1-year post-loading

## Results

### Clinical parameters

The plaque index was decreased and the gingival index was increased, indicating good plaque control measures during the course of the study. The variation of keratinized mucosa index, soft tissue width, and height at mesial, distal, and mid-buccal site were observed using stent pre-operative and at 6 months postoperative. The changes in soft tissue were statistically nonsignificant throughout the study (Table 1).

**Table 1:** Statistical analysis of variation clinical parameters

			Baseline		Post-op 6 months		Mean difference	*P* value significance
			Mean	SD	Mean	SD		
Soft tissue measurement using stent	Vertical	Mesial	6.75	1.5	6.94	1.3	− 0.19	0.19 NS
		Mid-buccal	8.63	1.8	8.6	1.81	0.031	0.92 NS
		Distal	7.16	1.65	7.38	1.31	− 0.22	0.443 NS
	Horizontal		2	0.41	2.13	0.5	− 0.13	0.41 NS
	Vertical	Mid-buccal	11.16	1.73	13.03	2.2	− 1.9	0.013S
Marginal bone level using stent		Mid-palatal/lingual	11.75	2.5	12.5	2.5	− 0.75	0.4 S
		Mid-mesial	11.4	1.6	12.0	1.6	− 0.56	0.3253 NS
		Mid-distal	11.4	1.72	12.0	1.7	− 0.6	0.34 NS
Using stent	Horizontal	Bucco-lingual/palatal	7	1.11	5.9	1.35	1.10	0.019 S
Radiographic bone level		Mesial	1.25	1.06	1.69	1.19	− 0.438	0.14 NS
		Distal	1.28	1.06	1.47	1.08	− 0.18	0.083 NS
ISQ value			69.13	4.22	71.31	4.50	2.18 ± 0.28	0.000 S
Crestal bone level (loading and 1 year post-op)			0.83	0.30	1.06	0.27	0.23 ± 0.03	0.000 S

Similarly, the variation of marginal bone level height and width were determined using a stent at the baseline and at 6 months postoperatively. On the mid-buccal site, at the baseline mean was 11.16 ± 1.73 mm, and at 6 months, it increased to 13.03 ± 2.2 mm with a mean difference of − 1.9 mm which was statistically significant (*p* = 0.013). The horizontal width mean of the marginal bone at the baseline was 7 ± 1.11 mm while it decreased to 5.9 ± 1.35 mm at 6 months with a mean difference of 1.10 mm which was statistically significant (*p* = 0.019). The radiographic parameters which evaluated the post-extraction bone changes were statistically not significant throughout the course of the study. For intragroup variation, Wilcoxon’s signed-rank test and paired *t* test were performed.

**Fig. 3:**
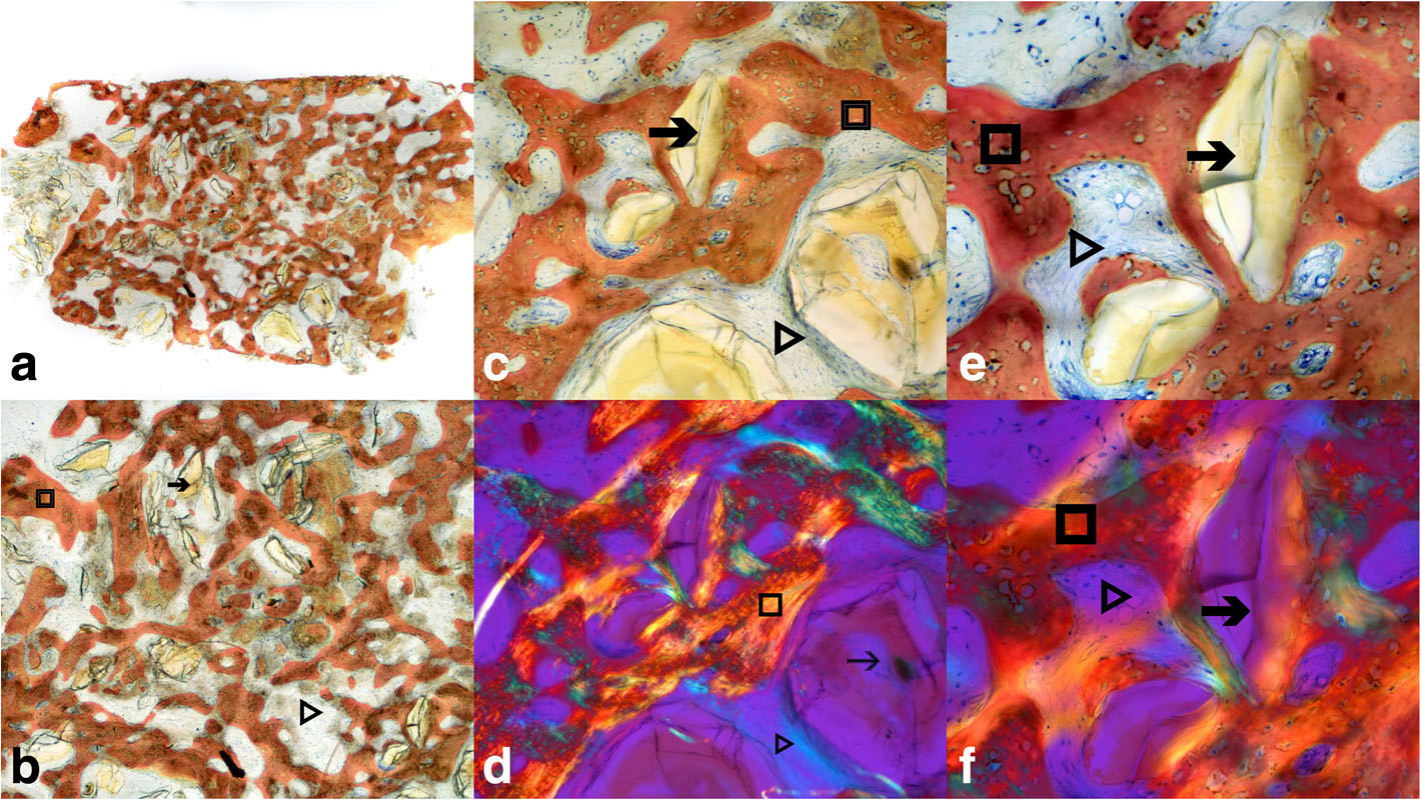
Histologic picture of harvested core in low × 10 (**a**), medium × 20 (**b**), high × 40 (**c**, **d**), and very high × 100 (**e**, **f**) magnifications under direct (**a**–**e**) and polarized (**d**, **f**) light. Histological picture of undecalcified section reveals vital bone lined by osteoblasts and embedded osteocytes (square-shaped marker). The amorphous areas of graft material surrounded by the vital bone are evident with collagenous tissue in-between. The trabecular spaces stained blue are seen with vascular elements (triangle-shaped marker). The new bone growth adherent to graft suggests the facilitation of bone growth by graft. The lack of inflammation suggests acceptance/absence of rejection of graft material (arrow-shaped marker) by the host bone

On the histomorphometric analysis, the cores revealed a new bone formation in all the grafted sockets. The mean percentage of the vital bone volume was 54.5 ± 16.76, non-mineralized tissue or marrow spaces 43.50 ± 15.80, and residual material 2.00 ± 3.37 (Fig. 3a–f).

### Implant assessment

**Fig. 4:**
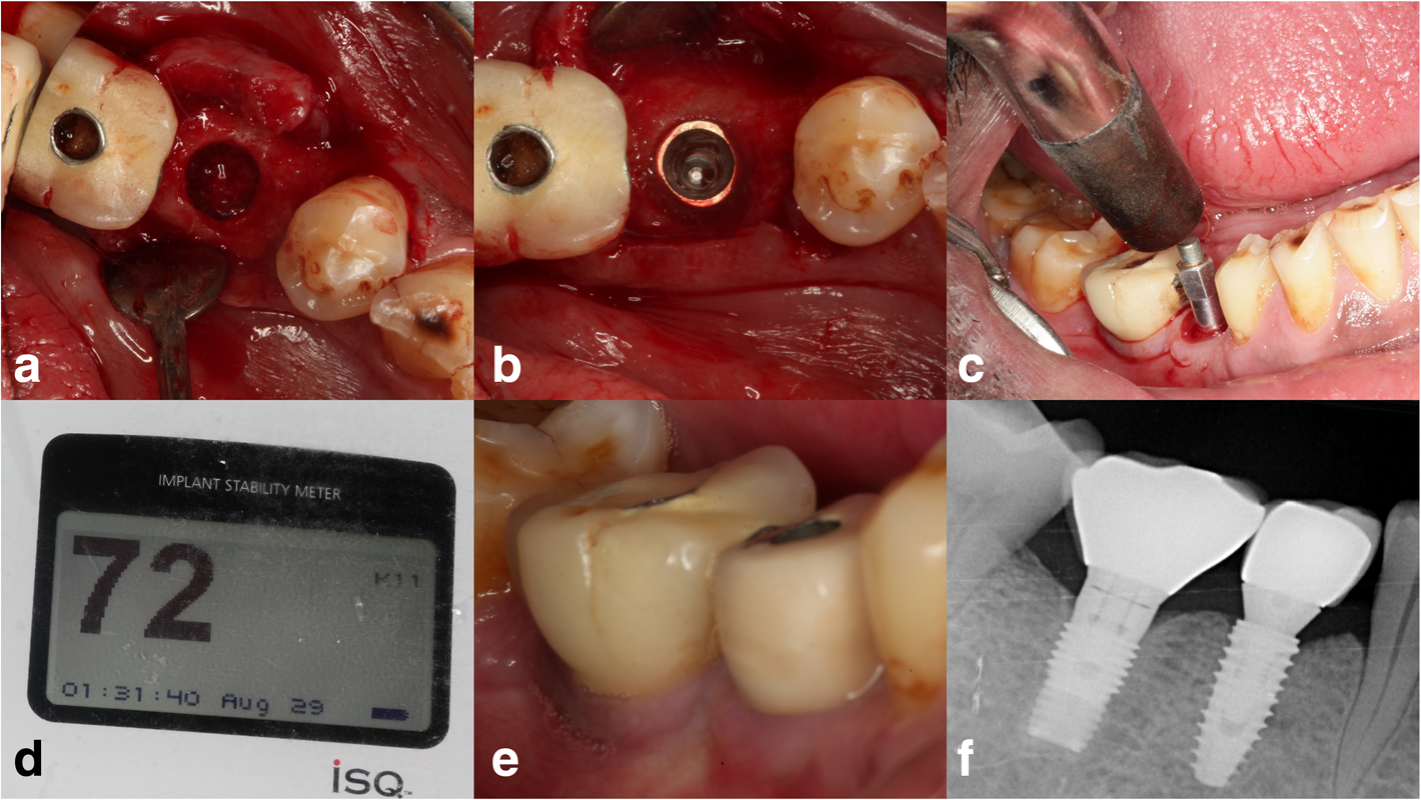
The healed socket after core harvesting (**a**) and implant placement (**b**). The stability implant placed is measured using smart peg in ISQ units (**c**, **d**). The clinical and radiographic appearance of prosthesis delivered with 1-year follow-up (**e**, **f**)

The ISQ values were calculated during the placement and loading (4 months after placement) [14]. The mean difference in ISQ values was 2.18 ± 0.28 which was statistically significant (*p* = 0.00). The crestal bone level with respect to implants was calculated on each implant during the loading and 1-year post-loading using intraoral periapical radiographs. The mean difference was 0.23 ± 0.03 which was statistically significant (*p* = 0.00) (Fig. 4a–f).

## Discussion

Araujo and Lindhe concluded and stated that “leaving the periosteum in place decreases the resorption rate of the extraction socket.” [15] Benex extraction system, a time-tested atraumatic extraction system, was thus incorporated in this study [4, 16, 17]. This calcium phosphosilicate putty exhibits an interesting property called osteostimulation apart from osteoconduction. On contact with body fluids, there is an immediate exchange of ions which results in a physiochemical bond between the bioglass, soft tissue, and bone. This results in the formation of a hydroxyl-carbonate apatite (HCA) layer, a biological apatite identical to the mineral phase of the bone, which allows for more rapid repair and regeneration of the bone than other synthetic graft materials [18, 19]. This alloplast stimulates the genes that control osteoblast differentiation and proliferation [20, 21]. Besides these, its ease of application, versatility, and usage like containment in the socket and ability to soften under pressure while loading makes the material more user-friendly. According to Pietrokovski, dense trabecular bone was formed in extraction sockets [22]. After bone grafting, PRF, a second-generation platelet concentrate, with its unique preparation technique, allows trapping of at least 95% of the platelets of the collected blood into a fibrin mesh which can then be easily manipulated into a membrane and transferred to any surgical site for the slow release of growth factors (GFs) from the platelet granules [23]. After PRF placement, a collagen plug was placed for the protection of blood clot, exclusion of gingival connective tissue, and provision of a secluded space into which osteogenic cells can migrate which are vital for bone regeneration [24]. Stabilization of the collagen plug atop the grafted bone was achieved by mattress suture. In this study, the postoperative healing of all the patients was uneventful and no complications were reported.

The combination of biomaterials used in this study satisfies the principles of bone regeneration. Primary wound closure was attained with atraumatic flapless extraction, angiogenesis was induced by autologous PRF placed, and space maintenance was achieved by the osteoconductive property of calcium phosphosilicate while the stability of the wound could be attributed to the collagen plug and mattress sutures placed after grafting [6].

On the histomorphometric evaluation of the bone core samples obtained at 6 months postoperative from the baseline, the overall mean value of the newly formed vital bone area fraction was 54.5 ± 16.76%. The formation of new well-mineralized vital trabecular bone was found in all the examined sections. The new bone was organized in trabeculae, with collagen fibers arranged in a meshwork pattern and osteocytes randomly distributed within the trabeculae in large spindle-shaped lacunae. These findings are in agreement with the previous studies [9, 25–27]. The 6 months post-grafting histomorphometric results of our study are parallel to the results of the meta-analysis evaluating the alloplast-mediated regeneration in extractions sockets [28]. A similar study was recently published wherein the soft tissue parameters pertained to healing index, while this study had standardized the parameter measurements using an acrylic stent with metallic wire which was preserved throughout the study period, for accurate measurements [29].

The 1-year success rate of the 262 implants placed in sockets grafted with calcium phosphosilicate putty was is 98.1% in a recent retrospective study [30]. Our study presents a 100% survival rate of implants, with predictable implant stability quotient values even after 1-year post-loading. Around 87% of implants exhibited an ISQ value above 65 which states that the quality of the augment bone is of type 1 [31]. The level of crestal bone around implants was minimal and said to be consistent with that of Kim et al. 2015 [32].

## Conclusion

Calcium phosphosilicate putty enriched with PRF and collagen plug is a predictable material for alveolar ridge preservation procedures. All the implants placed in these grafted sites were successful with acceptable stability which states this biomaterial could be considered as one of the novel grafts available with advantages of ease of handling and liable results. More studies with long-term follow-up are to be invited and comparative studies with gold standard materials are to be encouraged to provide foolproof data on different bone grafts and bone substitute materials.

## Data Availability

The clinical data and material required for the study were secured from the outpatient Department of Periodontics, Bapuji Dental College and Hospital.

## Abbreviations

%: Percentage
®: Recognized
ASA: American Society of Anesthesiologists
GF: Growth factor
HCA: Hydroxyl-carbonate apatite
HCl: Hydrochloride
ISQ: Implant stability quotient
PRF: Platelet-rich fibrin
UNC: University of North Carolina
